# Evaluation of a Digital Health Initiative in Illicit Substance Use: Cross-sectional Survey Study

**DOI:** 10.2196/29026

**Published:** 2021-08-10

**Authors:** Steph Kershaw, Louise Birrell, Hannah Deen, Nicola C Newton, Lexine A Stapinski, Katrina E Champion, Frances Kay-Lambkin, Maree Teesson, Cath Chapman

**Affiliations:** 1 The Matilda Centre for Research in Mental Health and Substance Use University of Sydney Sydney Australia; 2 Priority Research Centre for Brain and Mental Health The University of Newcastle Newcastle Australia

**Keywords:** methamphetamine, eHealth, substance-related disorders, internet, preventive psychiatry, health education, mobile phone

## Abstract

**Background:**

The *Cracks in the Ice* (CITI) community toolkit was developed to provide evidence-based, up-to-date information and resources about crystal methamphetamine to Australians. Given the high rates of internet use in the community and the potential for misinformation, CITI has the potential to play an important role in improving knowledge and challenging misconceptions surrounding crystal methamphetamine.

**Objective:**

This study aims to determine (1) whether the CITI toolkit is achieving its aim of disseminating evidence-based information and resources to people who use crystal methamphetamine, their family and friends, health professionals, and the general community and (2) examine the association between the use of CITI and the knowledge and attitudes about crystal methamphetamine.

**Methods:**

A cross-sectional web-based survey, open to Australian residents (aged ≥18 years), was conducted from November 2018 to March 2019. People who had previously visited the website (referred to as “website visitors” in this study) and those who had not (“naïve”) were recruited. At baseline, knowledge, attitudes, and demographics were assessed. CITI website visitors then completed a series of site evaluation questions, including the System Usability Scale (SUS), and naïve participants were asked to undertake a guided site tour of a replicated version of the site before completing the evaluation questions and repeating knowledge and attitude scales.

**Results:**

Of a total 2108 participants, 564 (26.7%) reported lifetime use of crystal methamphetamine, 434 (20.6%) were family/friends, 288 (13.7%) were health professionals, and 822 (38.9%) were community members. The average SUS score was 73.49 (SD 13.30), indicating good site usability. Health professionals reported significantly higher SUS scores than community members (*P*=.02) and people who used crystal methamphetamine (*P*<.001). Website visitors had significantly higher baseline knowledge than naïve participants (*P*<.001). Among naïve participants, knowledge scores increased following exposure to the website (mean 15.2, SE 0.05) compared to baseline (mean 14.4, SE 0.05; *P*<.001). The largest shifts in knowledge were observed for items related to prevalence, legal issues, and the effects of the drug. Stigmatizing attitude scores among the naïve group were significantly lower following exposure to CITI (mean 41.97, SE 0.21) compared to baseline (mean 44.3, SE 0.21; *P*<.001).

**Conclusions:**

This study provides an innovative evaluation of a national eHealth resource. CITI is achieving its aim of disseminating evidence-based, nonstigmatizing, and useful information and resources about crystal methamphetamine to key end user groups and has received good usability scores across its target groups. Interaction with CITI led to immediate improvements in knowledge about crystal methamphetamine and a decrease in stigmatizing attitudes. CITI demonstrates the important role of digital information and support platforms for translating evidence into practice and improving knowledge and reducing stigma.

## Introduction

### Background

Internationally, there continues to be a widespread concern about the use of methamphetamine, particularly with respect to the most potent form, crystal methamphetamine. Crystal methamphetamine produces stronger and longer-lasting effects than other forms of methamphetamine, thereby increasing the risk of serious long-term health issues, including dependence [1,2]. The use of crystal methamphetamine has been associated with considerable harms not only in individuals who use the drug but also in their families and communities [3]. Crystal methamphetamine has attracted a high level of negative attention compared with other drugs, particularly in Australia. In the 2019 National Drug Strategy Household Survey, the Australian community ranked meth/amphetamines as the drug of most serious concern and the drug most likely to be associated with a *drug problem* [4]. Its public ranking overtook alcohol in 2016 for the first time in the history of the survey [5]. Concerningly, crystal methamphetamine use has been frequently labeled as a national *crisis* or *epidemic*, with people who use crystal methamphetamine being negatively stereotyped in the media and being framed as criminal, deviant, or dangerous [6]. This is despite the prevalence of methamphetamine use being low, with only 1% of Australians reporting use in the previous 12 months [4].

In response to growing concern and negative attention around the drug, the Australian Government’s Department of Health funded the development of the *Cracks in the Ice* (CITI) community toolkit as an easily accessible, evidence-based, and up-to-date information resource [7]. The website was launched in 2017 and includes information about the prevalence, effects of the drug, treatment options, and support programs as well as tailored resources for families or friends, health professionals, and the community. The codevelopment process of CITI was broad-reaching and iterative, with the involvement of more than 450 community members across Australia (all states and territories), including people with lived experience, their families and friends, health professionals, and researchers [8]. CITI currently has 107 resources including factsheets, guidelines, animations or videos, and training and support programs and has reached more than 625,000 unique end users from across the globe (70% from Australia) with more than 1.2 million page views (Google Analytics as of March 22, 2021) and 2 national awards (Rotary Health Australia, 2017; The Mental Health Services Learning Network, 2020). A companion smartphone app, which allows users to access content offline, was launched in January 2018 to extend the reach and engagement of the toolkit, particularly for people in areas where internet access may be unreliable (ie, remote or regional areas) [9]. This is essential in a geographically diverse country such as Australia, where several data sources have indicated that the use of methamphetamine is higher, access to face-to-face services is lower, and unreliable internet connections are more common in regional and remote areas than in urban areas [10-12].

The internet has become a leading source of health information for the public, with eHealth platforms such as CITI offering a high level of accessibility and potential reach [13]. eHealth programs and websites have demonstrated an increase in empowerment and knowledge among health seekers on the internet [14] along with improved quality of care through the translation of evidence-based research into practice [15,16]. In Australia, it is estimated that 86.5% of the population are internet users, and 80% of Australians reported using the internet to search for health information in 2019 [17]. Indeed, during the development of CITI, the most commonly endorsed reasons given by participants for visiting a website about crystal methamphetamine were to “seek information for myself” (41.0%) and “to find out how to get help for a friend or a family member” (30.2%) [8]. However, there are risks associated with using the internet as a source of information, with many websites containing low-quality, irrelevant, inaccurate, or inappropriate health information [18,19]. Thus, a rigorous methodology is needed for both the development and evaluation of websites and eHealth resources. However, a recent systematic review found that although there are many toolkits for digital health, evaluations of these toolkits are uncommon [20]. This study aims to fill this gap. This evaluation is also one of the first to examine the relationship between knowledge and its impact on attitudes, an element not often looked at in website evaluations.

**Figure 1:**
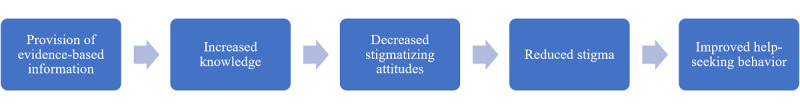
Proposed conceptual model of how providing evidence-based information can lead to increased knowledge and decreased stigma and ultimately improve help-seeking behaviors among people who use crystal methamphetamine.

Given the large proportion of Australians who use the internet, the potential for misinformation, and the high level of interest in crystal methamphetamine use, initiatives such as CITI play an important role in not only disseminating accurate evidence-based information but also reducing the stigma and discrimination surrounding crystal methamphetamine use. Recent research by our team indicates that stigma toward people who use crystal methamphetamine is common in Australia, with 1 in 3 people who use crystal methamphetamine reporting that they have been discriminated against because of their drug use [21]. Stigma is a source of immense psychological distress and has been associated with feelings of self-blame, low self-worth, shame, and higher rates of drug dependence and lower rates of treatment seeking for substance use problems and other health conditions such as mental illness, trauma, and infections [22-24]. Importantly, our previous study also demonstrated that higher levels of accurate knowledge about crystal methamphetamine were associated with less stigmatizing attitudes [21]. Thus, by improving knowledge about crystal methamphetamine, CITI has the potential to influence public attitudes toward those affected by the drug and support people to seek help when they need it (Figure 1).

### Objectives

This study presents a large community evaluation of an innovative centralized eHealth resource for evidence-based information and resources on crystal methamphetamine. The aims of this study were to (1) determine whether the CITI toolkit is achieving its aim of disseminating evidence-based, nonstigmatizing, and useful information and resources about crystal methamphetamine to key end user groups (people who use crystal methamphetamine, affected family members and friends, health professionals, and the general Australian community) and (2) examine the association between the use of CITI and improvements in knowledge and attitudes about crystal methamphetamine and harms.

## Methods

### Design and Procedure

Between November 2018 and March 2019, participants were recruited via convenience sampling methods, including advertising on the CITI website, Facebook page, Twitter page, e-newsletter, and through paid public advertising on Facebook. The eligible participants provided informed consent before completing a web-based cross-sectional survey that was open to all Australian residents aged ≥18 years. At the end of the survey, all participants were given the opportunity to provide their email address to enter a draw to win an Apple iPad. Ethics approval was obtained from the University of New South Wales (HC# HC180735) and the University of Sydney (Project 2018/844) human research ethics committees.

**Figure 2:**
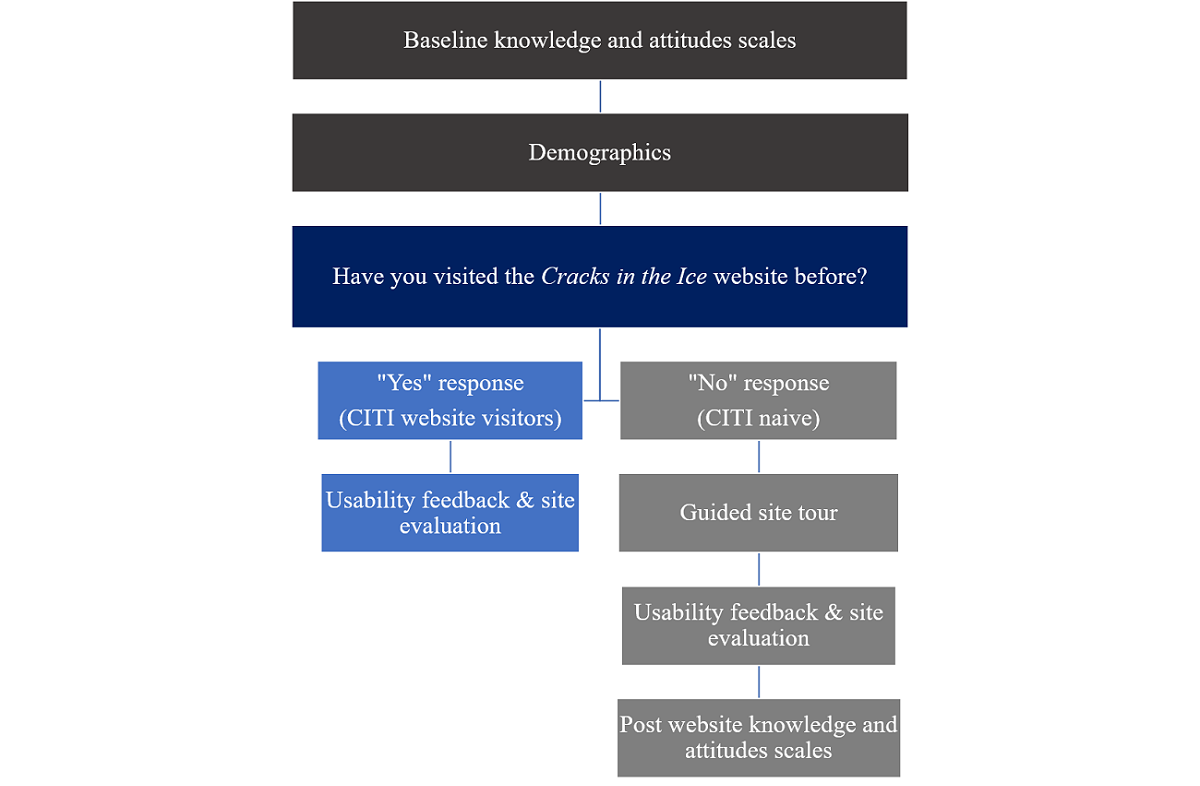
Flow chart of the study design. CITI: *Cracks in the Ice*.

To address both aims of the study, people who had previously visited the website (CITI website visitors) and people who had not previously visited the website (CITI naïve) were recruited (Figure 2). At baseline, all participants completed a series of knowledge and attitude scales, along with standard demographic questions. Participants were then divided into 2 groups depending on their answer to the question “Have you visited the CITI website before?” (yes, no, or unsure). CITI website visitors completed a series of site evaluation questions, whereas CITI-naïve participants were asked to undertake a guided site tour of a replicated version of the live CITI site before completing the site evaluation questions and repeating the knowledge and attitude scales. This methodology permitted the assessment of any change in these constructs associated with viewing the website. As a part of the guided site tour, CITI-naïve participants were encouraged to view the homepage and key webpages featuring content on the prevalence and physical and mental health effects of crystal methamphetamine use along with treatment and support options. As CITI is a live responsive website, the entire site was replicated to allow measurement of usage behavior by study participants. Participants who selected *unsure* were assigned to the CITI-naïve group; however, they were excluded from group comparison analyses to reduce any possible confounding of results.

### Measures

Information collected included age (in years), gender, residential postcode, and region (metropolitan, regional, or rural or remote). Participants were asked to identify whether they had previously used crystal methamphetamine, had a family member or friend who used (or who they thought might be using) crystal methamphetamine, or were a health professional. All participants were asked to indicate whether they had previously viewed the CITI website.

The System Usability Scale (SUS) is a reliable scale for measuring usability, including 10 items about several facets such as the complexity of the website and ease of use. The 10 items were scored on a 5-point Likert scale ranging from 0 (strongly disagree) to 5 (strongly agree), with scores calculated as validated in the literature [25]*.* Scale scores ranged from 0 to 100, with higher scores indicating enhanced usability. The average SUS score based on more than 500 studies was 68, with scores of 80 or above indicating a strong performance [26].

Participants also responded to an additional 4 items about the website’s purpose, goals, and evidence base adapted from previous research by our team [8] with items being (1) “The support options listed on *Cracks in the Ice* are useful,” (2) “The primary goal/purpose of *Cracks in the Ice* is clear,” (3) “The information and resources on *Cracks in the Ice* have been informed by evidence,” and (4) “The terminology on *Cracks in the Ice* is nonstigmatizing.” These statements were scored on a 5-point Likert scale, ranging from 0 (strongly disagree) to 5 (strongly agree). In addition, participants were asked on a 7-point Likert scale, ranging from 0 (strongly disagree) to 7 (strongly agree), “I am confident in helping someone who has a problem with ice” and on a 5-point Likert scale, “If I knew someone who was using ice, I would help them.” Participants were given the opportunity throughout the questionnaire to provide open-ended feedback about the website. The term “ice” was used instead of “crystal methamphetamine” as it is a more commonly known term for crystal methamphetamine among the Australian community.

To assess knowledge of crystal methamphetamine, participants completed a series of 18 *true or false* questions (each with three response options: 1=true, 2=false, and 3=unsure). Questions included knowledge of its effects on the brain and body (eg, “Ice will cause psychosis in all people”), rates of use (eg, “Methamphetamines [including ice] are the most popular illicit drugs in Australia”), and legal status in Australia. The total knowledge score was calculated by summing the number of correctly answered items (min=0 and max=18). *Unsure* responses were coded as incorrect for the purposes of analysis. This measure was developed by the authors as part of the development of CITI to assess key areas of knowledge and potential misconceptions about crystal methamphetamine among a lay audience [8] and has been used in other analyses by our group [21].

To assess attitudes toward people who use methamphetamine, participants responded to a 14-item, 5-point Likert scale ranging from 1 (strongly disagree) to 5 (strongly agree). Ten items were adapted from the Brener and Von Hippel scale to measure attitudes toward people who inject drugs. The reliability and validity of the original scale have been previously demonstrated [27]. Mentions of “people who inject drugs” in the original scale were replaced with “people who use ice.” Four additional items were adapted from the Depression Stigma Scale (DSS) with mention of mental health issues being replaced with “people who use ice” to assess perceptions of personal blame or control (“A person using ice could recover if they just stopped using” or “Using ice is a sign of personal weakness”), danger (“People who use ice are dangerous”), and shame (“If I had a problem with ice, I would not tell anyone”) [28]. Although the DSS was originally designed to measure stigma attached to depression, it has also been used to assess stigma attached to other disorders [29,30]. The reliability and validity of the DSS have been demonstrated in prior studies [28,30]. Positive statements were reverse coded before the total score was calculated (min=14 and max=70), with higher scores indicating more negative attitudes. The adapted scale demonstrated good internal consistency in the current sample (Cronbach α=.86).

### Data Analysis

Data were analyzed using the IBM SPSS Statistics version 24. Analysis of variance was conducted to assess differences in system usability scores of end user groups and the impact of CITI on knowledge and attitudes about crystal methamphetamine. Chi-square tests were used to compare ratings on Likert scale statements among the 4 end user groups, with “strongly disagree” and “disagree” ratings combined for the purpose of analyses, as the count was too low among the health professional end user group. Two-tailed *t* tests were also conducted to assess whether knowledge was different between the CITI website visitors and CITI-naïve groups and within the CITI-naïve group before and after visiting the CITI website. Content analysis of open-ended feedback using general inductive analysis [31] was also performed to identify core themes.

## Results

### Participants

A total of 2125 participants completed the survey. Among them, 17 participants were excluded from the analysis owing to invalid responses (eg, rapid responding or inconsistent responses), leaving a total of 2108 participants in the final sample. The mean age was 36 (SD 13) years, and 58.6% (1235/2108) of the participants identified as female. More than half of the participants were from metropolitan areas (1190/2108, 56.5%), a third (691/2108, 32.8%) were from regional locations, and 10.8% (227/2108) were from rural or remote locations. The sample’s age, gender, and state or territory of residence aligned closely with national population statistics from the 2018 Australian Bureau of Statistics [32]. A total of 26.7% (564/2108) of participants reported lifetime use of crystal methamphetamine (any use of the drug either current or in the past). Table 1 summarizes the descriptive statistics of gender, age, and region for each of the target end user groups (people who use crystal methamphetamine, affected family and friends, health professionals, and the general Australian community). The respondents in the “people who use crystal methamphetamine” group included both people who were currently using and those who reported using it in the past.

**Table 1:** Demographics by end user groups (N=2108).

Demographics		End user group			
		People who use crystal methamphetamine (n=564)	Family and friends (n=434)	Health professionals (n=288)	Community members (n=822)
**Gender, n (%)**					
	Female	278 (49.3)	306 (70.5)	212 (73.6)	439 (53.4)
	Male	270 (47.9)	113 (26.0)	75 (26.0)	368 (44.8)
	Nonbinary or gender fluid	5 (0.9)	11 (2.5)	0 (0)	13 (1.6)
	Different identity or prefer not to say	11 (1.9)	4 (1.0)	1 (0.3)	2 (0.2)
Age (years), mean (SD)		35.76 (9.42)	39.45 (14.55)	40.42 (12.72)	33.57 (13.64)
**Region, n (%)**					
	Metro	343 (60.8)	215 (49.5)	146 (50.7)	486 (59.1)
	Regional	176 (31.2)	161 (37.1)	92 (31.9)	262 (31.9)
	Rural or remote	45 (8.0)	58 (13.4)	50 (17.4)	74 (9.0)

### Usability and Site Evaluation

The average SUS score for the CITI website was 73.49 (SD 13.30), indicating good usability. Table 2 provides the average SUS score for each end user group. SUS scores were significantly higher in the health professionals group than in the community (*F*_3,2062_=6.8; *P*=.02) and people who use crystal methamphetamine (*F*_3,2062_=6.8; *P*<.001) end user groups. SUS scores were also significantly higher in the family and friends group than in the people who used crystal methamphetamine group (*F*_3,2062_=6.8; *P*=.04).

**Table 2:** System Usability Scale scores for each end user group (N=2066)^a^.

End user group	Participants, n (%)	SUS^b^, mean (SD)
Community members	809 (39.1)	73.53 (13.43)
People who use crystal methamphetamine	553 (26.7)	71.73 (13.31)
Family and friends	419 (20.2)	74.12 (13.92)^c^
Health professionals	285 (13.7)	75.91 (11.41)^d^

^a^A total of 42 participants were excluded from the analyses because they selected unsure when asked if they had visited the *Cracks in the Ice* website.

^b^SUS: System Usability Scale.

^c^*P*=.02.

^d^*P*<.01.

**Figure 3:**
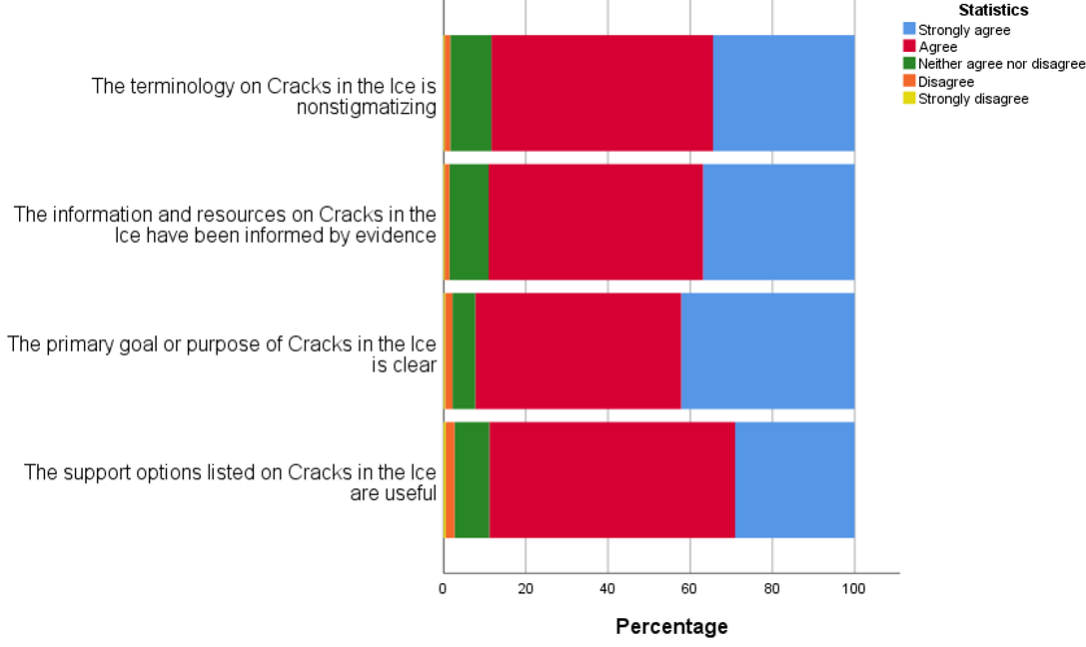
Participant feedback of the *Cracks in the Ice* website’s purpose, goals and, evidence base on a 5-point Likert scale.

Participants had an overall positive response to the website’s purpose, goals, and evidence base (Figure 3), with most participants providing a rating of *agree* or *strongly agree* for statements such as “The information and resources has been informed by evidence” (1839/2066, 87.2%), “The terminology is nonstigmatizing (1825/2066, 86.6%), “The primary goal/purpose is clear” (1907/2066, 90.5%), and “The support options listed are useful” (1836/2066, 87.1%).

Ratings for each statement were compared, with significant associations observed between the end user group and ratings for each of the statements (χ^2^_9_ range 43.79-67.05; *P*<.001). Agreement was generally higher among the community, health professional, and family and friends groups compared with people who use crystal methamphetamine; however, the differences were small (between 5% and 12%). Further descriptive information for each statement by the end user group is detailed in Multimedia Appendix 1.

Open-ended comments left by survey respondents at the end of the survey were mostly positive, stating that the website was evidence-based, informative, easy to use, and presented information in a nonjudgmental or nonstigmatizing way. Other comments related to improving the user experience or navigation, creating more resources such as lived experience stories, and specific resources to support regional or rural areas along with Aboriginal and Torres Strait Islander peoples. Several examples of the comments are provided in Multimedia Appendix 2.

### Knowledge and Attitudes About Crystal Methamphetamine and Harms

#### Overview

There were 277 participants who had previously visited the website (CITI website visitors) and 1789 participants who had not previously visited the website (CITI-naïve group). An additional 42 participants were excluded from further analyses as they selected the response *unsure* when asked if they had visited the website.

The CITI-naïve group spent an average of 2.16 minutes on the replicated website, visiting 2.17 pages, spending an average of 82.63 (SD 189.5) seconds on relevant content pages (Google Analytics November 19, 2018, to March 31, 2019). This is in line with the average session duration of between 2 and 3 minutes for large websites, such as many health care sites [33]. Previous experience with the website resulted in significantly higher SUS scores in the CITI website group (*t*_1_=8.36; *P*=.004) compared with the CITI-naïve group.

On average, CITI website visitors had higher total baseline knowledge scores (mean 15.2, SE 0.1) than those who had not previously visited the website (mean 14.4, SE 0.05) (Table 3). This difference was significant (t_2064_=5.54; *P*<.001), with a small to medium–sized effect (Cohen *d*=0.44).

**Table 3:** Comparison of knowledge between *Cracks in the Ice* (CITI) visitors and the CITI-naïve group at baseline, indicating the numbers and percentages of participants who gave correct answers to the knowledge statement (N=2108)^a^.

Knowledge statement	Total sample (n=2108), n (%)	CITI^b^ visitors (n=277), n (%)	CITI-naïve (n=1789), n (%)
It is illegal to drive while under the influence of ice	2069 (98.1)	271 (97.8)	1756 (98.15)
Ice use can lead to serious long-term physical effects	2056 (97.5)	270 (97.5)	1745 (97.54)
Using ice can affect your sleep	2019 (95.8)	273 (98.6)	1705 (95.30)
Ice use can make you feel paranoid	2017 (95.7)	272 (98.2)	1705 (95.30)
Ice use can make you feel agitated or distressed	2013 (95.5)	272 (98.2)	1701 (95.08)
You always know what you’re taking when you use ice	1962 (93.1)	259 (93.5)	1663 (92.95)
Taking ice with other drugs can reduce the risks of harm	1910 (90.6)	253 (91.3)	1620 (90.55)
Low doses of ice will not impair driving skills	1908 (90.5)	258 (93.1)	1611 (90.05)
It is legal to share ice with friends	1818 (86.2)	238 (85.9)	1544 (86.30)
It is impossible to break an ice dependence	1771 (84.0)	249 (89.9)	1490 (83.28)
The effects of ice are short-lived	1731 (82.1)	235 (84.8)	1464 (81.83)
Ice is generally more potent (strong) than speed	1718 (81.5)	239 (86.3)	1445 (80.77)
You can go to jail for using methamphetamines, including ice	1578 (74.9)	215 (77.6)	1334 (74.56)
Most teenagers have used ice	1570 (74.5)	220 (79.4)	1325 (74.06)
Ice will cause psychosis in all people	1408 (66.8)	216 (78.0)	1172 (65.51)
Legal penalties for drug offences relating to methamphetamine in Australia are different in each of the states and territories	1310 (62.1)	196 (70.8)	1087 (60.76)
Methamphetamines (including ice) are the most popular illicit drugs in Australia	899 (42.6)	144 (52.0)	743 (41.53)
Most of the time, police will not be called when an ambulance is attending a drug overdose	808 (38.3)	126 (45.5)	668 (37.33)

^a^Statements are ordered from most to least correctly answered among the total sample.

^b^CITI: *Cracks in the Ice*.

#### Change in Knowledge and Attitudes After Viewing the Website

The CITI-naïve group was assessed on knowledge both before and after website viewing, with results showing that, on average, knowledge scores significantly increased following exposure to the website (mean 15.2, SE 0.05) compared with baseline (mean 14.4, SE 0.05; t_1788_=17.893; *P*<.001), as displayed in Table 4. The largest shifts in knowledge were observed for items related to the prevalence of crystal methamphetamine use, legal issues, and the physical and mental effects of the drug. There was a 35.1% change (n=261) for “Methamphetamines (including ice) are the most popular illicit drugs in Australia,” a 24.0% change (n=160) for “Most of the time, police will not be called when an ambulance is attending a drug overdose,” 22.0% change (n=239) for “Legal penalties for drug offences relating to methamphetamine in Australia are different in each of the states and territories,” 16.8% change (n=197) for “Ice will cause psychosis in all people,” and 10.8% change (n=161) for “It is impossible to break an ice dependence” following exposure to the CITI website.

**Table 4:** Knowledge among the *Cracks in the Ice*–naïve sample at baseline and after website exposure, indicating the number and percentage of people giving correct answers to knowledge statements (N=1789).

Knowledge statement	Baseline, n (%)	Postexposure^a^, n (%)
Methamphetamines (including ice) are the most popular illicit drugs in Australia	743 (41.53)	1004 (56.12)
Most teenagers have used ice	1325 (74.06)	1418 (79.26)
Ice is generally more potent (strong) than speed	1445 (80.77)	1561 (87.25)
You always know what you’re taking when you use ice	1663 (92.95)	1702 (95.13)
Using ice can affect your sleep	1705 (95.30)	1759 (98.32)
Taking ice with other drugs can reduce the risks of harm	1620 (90.55)	1660 (92.78)
Ice use can lead to serious long-term physical effects	1745 (97.54)	1757 (98.21)
It is impossible to break an ice dependence	1490 (83.28)	1651 (92.28)
You can go to jail for using methamphetamines, including ice	1334 (74.56)	1342 (75.01)
It is legal to share ice with friends	1544 (86.30)	1473 (82.33)
Most of the time, police will not be called when an ambulance is attending a drug overdose	668 (37.33)	828 (46.28)
It is illegal to drive while under the influence of ice	1756 (98.15)	1732 (96.81)
The effects of ice are short-lived	1464 (81.83)	1542 (86.19)
Low doses of ice will not impair driving skills	1611 (90.05)	1647 (92.06)
Ice will cause psychosis in all people	1172 (65.51)	1369 (76.52)
Ice use can make you feel agitated or distressed	1701 (95.08)	1761 (98.43)
Ice use can make you feel paranoid	1705 (95.30)	1756 (98.15)
Legal penalties for drug offences relating to methamphetamine in Australia are different in each of the states and territories	1087 (60.76)	1326 (74.11)

^a^Average knowledge scores increased following exposure to the website (mean 15.2, SE 0.05) compared to the baseline (mean 14.4, SE 0.05; t_1788_=17.893; *P*<.001).

On average, the total attitude score among the CITI-naïve group was significantly lower following exposure to the CITI website (mean 41.97, SE 0.21) compared with the baseline (mean 44.3, SE 0.21; t_1788_=23.91; *P*<.001), with lower scores indicating less negative or stigmatizing attitudes (Table 5). Follow-up analyses indicated that significant before and after changes in knowledge and attitude scores were observed in all 4 end user groups.

**Table 5:** Endorsement of attitudes toward people who use crystal methamphetamine of the *Cracks in the Ice*–naïve sample at baseline and after website exposure (N=1789)^a^.

Attitude statement		Baseline, n (%)	Postexposure^b^, n (%)
**Negative attitudes**			
	I won’t associate with people who use ice if I can help it	1233 (68.92)	986 (55.11)
	I avoid people who use ice whenever possible	1232 (68.88)	1103 (61.65)
	Use of ice is just plain wrong	986 (55.11)	863 (48.23)
	People who use ice are dangerous	821 (45.89)	588 (32.86)
	A person using ice could recover if they just stopped using	737 (41.19)	621 (34.71)
	If I had a problem with ice, I would not tell anyone	597 (33.37)	395 (22.07)
	Use of ice is immoral	470 (26.27)	408 (22.80)
	Using ice is a sign of personal weakness	251 (14.03)	182 (10.17)
	People who use ice should be locked up to protect society	241 (13.47)	173 (9.67)
**Positive attitudes**			
	People should feel sympathetic and understanding of people who use ice	821 (45.89)	924 (51.64)
	People who use ice are mistreated in our society	716 (40.02)	837 (46.78)
	People who use ice have a perfect right to their lifestyle, if that's the way they want to live	260 (14.53)	237 (13.24)
	People who use ice should be accepted completely into our society	251 (14.03)	317 (17.71)
	The use of ice is merely a different kind of lifestyle that should not be condemned	185 (10.34)	186 (10.39)

^a^Participant selected *agree* or *strongly agree* on 5-point Likert scale.

^b^Total attitude score was significantly lower following exposure to the *Cracks in the Ice* website (Mean 41.97, SE 0.21) compared with the baseline (mean 44.3, SE 0.21; t1788=23.91; *P*<.001).

In addition, among the CITI-naïve group, exposure to the CITI website resulted in minor improvements (eg, selecting neutral or agree responses) to the two additional statements “If I knew someone who was using ice, I would help them” and “I am confident in helping someone who has a problem with ice” (Table 6).

For the “If I knew someone who was using ice, I would help them” statement, measured on a 7-point Likert scale from strongly disagree to strongly agree, 25.1% (n=448) increased their rating, 16.3% (n=292) decreased their rating, whereas 58.6% (n=1049) made no change in their rating after viewing the website. For the statement “I am confident in helping someone who has a problem with ice,” measured on a 5-point Likert scale from strongly disagree to strongly agree, 44.1% (n=790) reported an increase in rating, 3.6% (n=64) decreased their rating, whereas 52.3% (n=935) made no change in their rating after viewing the website.

**Table 6:** Rates of agreement with helping statements before and after website exposure among *Cracks in the Ice*–naïve group (N=1789).

Statement		Baseline, n (%)	Postexposure, n (%)
**If I knew someone who was using ice, I would help them**			
	Strongly disagree	33 (1.8)	30 (1.7)
	Mostly disagree	47 (2.6)	47 (2.6)
	Somewhat disagree	65 (3.6)	33 (1.8)
	Neither agree nor disagree	151 (8.4)	140 (7.8)
	Somewhat agree	490 (27.4)	479 (26.8)
	Mostly agree	561 (31.4)	543 (30.4)
	Strongly agree	442 (24.7)	517 (28.9)
**I am confident in helping someone who has a problem with ice**			
	Not at all	543 (30.4)	191 (10.7)
	A little bit	448 (25.0)	448 (25.0)
	Moderately	451 (25.2)	596 (33.3)
	Quite a bit	234 (13.1)	399 (22.3)
	Extremely	113 (6.3)	155 (8.7)

## Discussion

### Principal Findings

This study evaluated CITI*,* the first centralized Australian eHealth resource to provide information and resources about crystal methamphetamine to support the community [8]. This evaluation is one of the most rigorous evaluations of eHealth resources to date, conducted among a large sample of more than 2000 Australians. This website evaluation was also one of the first studies to examine the effect of an eHealth resource on knowledge and attitudes regarding illicit substance use. Overall, the results indicated that CITI is achieving its aim of disseminating evidence-based, nonstigmatizing information and resources about crystal methamphetamine to key end user groups (people who use the drug, affected family members and friends, health professionals, and the general Australian community). This is evidenced by more than 80% of participants endorsing statements about the clarity of the website’s purpose, goals, and evidence base. Endorsement of the website’s purpose and goals was generally higher among the community, health professional, and family and friends groups compared with people who use crystal methamphetamine; however, differences were small (between 5% and 12%). It is a challenge for CITI to cater to different end user groups that have differing needs and has been addressed by providing user-specific sections in addition to general information. Therefore, it was important to check whether these key end user groups had been adequately catered for and identify any improvements that might be needed for the tailored resources.

The average SUS score for CITI was 73.49 (SD 13.30), indicating good usability, which is above the average of 68, based on >500 studies [26]. An SUS of 80 or above is an indication of strong performance, and future iterations of CITI will aim to further improve user experience on the site to reach this level. The differences in SUS scores between different stakeholder groups is a key point of interest, with health professionals reporting significantly higher SUS scores than general community members or people who use crystal methamphetamine. This may be because of health professionals having high digital literacy levels [34] and having higher general levels of education. We also observed higher SUS scores among family and friends than among people who used crystal methamphetamine. This may reflect the fact that the support of families and friends was a priority area in site development, with fewer resources available for people who use crystal methamphetamine. Creating and tailoring information and resources for people who use crystal methamphetamine is an area of growth for the website, and several such resources are currently in development. Unsurprisingly, familiarity with the website (measured by visiting the website before the evaluation) was associated with significantly higher SUS scores.

The finding that the use of CITI led to improvements in knowledge and attitudes about crystal methamphetamine and harms in the naïve group, despite the average time spent on the replicated website being 2 minutes, is encouraging. The average knowledge scores significantly increased after viewing the site, although the effects were small. Examination of individual items showed some promising shifts, particularly for items that are often common misconceptions about the drug (eg, prevalence of use, legal issues, and the physical and mental effects of the drug). Thus, providing some support that the shifts could be meaningful in areas where knowledge is lacking or incorrect. These changes in knowledge are of particular salience given the misconceptions often portrayed in the Australian media about the methamphetamine *epidemic* and the framing of people who use the drug as criminal, deviant, or dangerous [6]. Unsurprisingly, those already familiar with the website before the study had higher total baseline knowledge scores. This may reflect the fact that they had previously reviewed the key informational pages or the possibility that those who had sought out the CITI website of their own volition previously had more knowledge about the drug.

The decrease in total attitude score among the CITI-naïve group (indicating less negative or stigmatizing attitudes) following exposure to the website is also promising. We have previously found that increased knowledge about the drug is associated with less stigmatizing attitudes [21]. This also aligns with what has been found in mental health research, where improvements in knowledge have led to increased positive attitudes or less stigmatizing beliefs [35-38]. Through CITI’s dissemination of evidence-based information about crystal methamphetamine, it has the potential to change public attitudes and reduce the stigma associated with the drug. This is potentially an important shift, given that stigma is consistently cited as one of the main barriers to care among people who use methamphetamine [39].

### Comparison With Previous Work

eHealth resources are a low-cost way to increase public awareness of important issues and have the potential to reach a large number of people [40]. However, they must respond to rapidly changing population trends and evidence. In areas such as new and emerging drugs, this is particularly important [41]. Therefore, it is important for eHealth resources to be living resources that are adequately funded to respond to emerging research evidence. Consumer health websites need to be not only evidence-based and trustworthy sources of information but also user friendly and meet the health information-seeking needs of their target groups [42,43]. Therefore, website evaluations commonly use a variety of methodologies, including measures of usability (SUS and task performance), self-reports (surveys), qualitative interview-based methods, or Google Analytics to investigate website behaviors (use and interaction) [44-47]. Similar to a recent study, we did not find published recommendations for procedures to conduct a review of a live website in a systematic manner [48]. A recent systematic review found that despite there being many published toolkits, very few were evaluated, and the authors concluded that this is an area where greater attention and rigorous methodology are needed [20]. Although review criteria [49] and a possible framework (Reach, Effectiveness, Adoption, Implementation, Maintenance) from implementation science [50] have been proposed as useful avenues for evaluation, they are more appropriate for web-based intervention–based studies on behavior change. Within this context, we note that this website evaluation is one of the most rigorous evaluations in the field and is a solid starting point, with the potential for future experimental designs. This evaluation also involved the examination of knowledge and its impact on attitudes, an element not often looked at in website evaluations.

It is generally assumed that the longer a visitor spends on a website, the more engaged they are. However, it is difficult to find or estimate a benchmark for the average time spent on health-related websites, as time spent visiting sites depends on the scope and purpose of the actual site. It has been reported that for large websites, such as many health care sites, the average session duration is between 2 and 3 minutes [33]. However, it is difficult to find a benchmark for comparison with public health websites such as CITI because of the large variability across sites and industries. In this study, the CITI-naïve group that undertook the site tour spent an average time of 2.16 minutes, visiting 2.17 pages, in line with the average session duration of health care sites. This is slightly higher than the average time for website users of the live website, which has an average session time of 1.42 minutes with people visiting an average of 1.65 pages (April 3, 2017, to December 31, 2020). It would be useful for future studies to examine the impact of time spent on the site and to determine whether strategies to increase the length of time users engage with the site would be useful to encourage knowledge or attitude change.

The CITI eHealth resource also caters to several different end user populations (eg, health professionals, community members, family and friends, and people who use crystal methamphetamine). As such, similar to other websites, challenges remain in distinguishing between and meeting the needs of different groups [40]. This was evident in this study with people who use crystal methamphetamine having slightly lower endorsement of the website’s purpose and goals. Therefore, more in-depth investigation is needed to understand the information needs of people who use crystal methamphetamine. Another avenue for future evaluation is to ensure that the information provided on CITI is suitable to people from diverse backgrounds and varying levels of health literacy, an important consideration that has been noted in the literature [51].

### Limitations

This study is not without its limitations, and its findings should be interpreted with these in mind. As CITI is a live website, which can be accessed at any time, this study used a quasi-experimental design. Future evaluations may consider creating beta versions of the site that could be tested in an A/B test with a control group. Another limitation is that although the CITI-naïve group was directed to specific pages during the site tour, there was no minimum time to stay on each page enforced, and participants could also review pages other than those specifically mentioned. Therefore, although changes in knowledge were significant, it is difficult to ascertain the direct cause of this change and whether, for example, it was related to the amount of time participants spent on the site or the specific pages they visited. Similarly, as this study is not longitudinal in its design, it is unclear whether these knowledge shifts are maintained over time or whether changes in knowledge and attitudes lead to behavior change.

This study used convenience sampling methods; therefore, the sample was not nationally representative. However, the sample’s age, gender, and state or territory of residence aligned closely with national population statistics from the Australian Bureau of Statistics [32]. Similarly, the use of paid Facebook advertising may have attracted individuals with an interest in the drug crystal methamphetamine; thus, the knowledge and attitude findings may not be representative of the general population.The use of paid advertising also resulted in the sample of CITI-naïve participants being 6 times larger than the group already familiar with CITI; however, the sample sizes achieved in each group were large for studies of this kind.

It should also be noted that the measure used to assess knowledge about crystal methamphetamine was developed by the research team in response to a lack of published measures for this purpose. As such, the psychometric properties of the measure are unknown, including whether knowledge as measured in this study is a multidimensional or unidimensional construct. Therefore, direct conclusions about improving which aspects of knowledge (or knowledge in general) might reduce stigmatizing attitudes cannot be made.

### Conclusions

This study is one of the most comprehensive and rigorous evaluations of a centralized national eHealth resource for the general public, focusing on substance use. CITI, an innovative portal for evidence-based information and resources about crystal methamphetamine, was found to achieve its aim of disseminating evidence-based, nonstigmatizing, and useful information and resources about crystal methamphetamine to key end user groups. Furthermore, it received good usability scores across its target groups and was found to be easy to use. In addition, interaction with CITI led to immediate improvements in knowledge and a decrease in stigmatizing attitudes about the drug, which is promising given the links among knowledge, stigma, and help-seeking. CITI demonstrates the important role of digital information and support platforms for translating evidence into practice as well as improving knowledge and reducing stigma around crystal methamphetamine. It is hoped that this will ultimately lead to an increase in help-seeking and evidence-based service use.

## Appendix

Multimedia Appendix 1 Ratings on Likert scale items about the purpose, goals, and evidence base of the website.

Multimedia Appendix 2 Examples of open-ended comments from the study participants.

## Abbreviations

CITI: Cracks in the Ice
DSS: Depression Stigma Scale
SUS: System Usability Scale
