# Supplementary material for: Evaluation of a Digital Health Initiative in Illicit Substance Use: Cross-sectional Survey Study
Source: J Med Internet Res. 2021 Aug 10;23(8):e29026. doi: 10.2196/29026 (PMC8386404; doi:10.2196/29026)
Supplement: Multimedia Appendix 1 [file jmir_v23i8e29026_app1.docx]

### Multimedia Appendix 1

### Supplementary Table S1: Ratings on Likert Scale items about website purpose, goals and evidence base.

**Table S1 a):** **Ratings for the statement “*The support options listed on Cracks in the Ice are useful*” broken down by end-user groups of Cracks in the Ice.** A significant association between group and ratings on the 5-point Likert Scale were observed (*Χ*^2^_9_ = 67.05, *P* <0.01).

|  | **Strongly agree** | **Agree** | **Neither agree nor disagree** | **Disagree/Strongly Disagree** |
| --- | --- | --- | --- | --- |
| Community (n=809) | 267 (33.0) | 483 (59.7) | 49 (6.1) | 10 (1.2) |
| People who use ice (n=553) | 109 (19.7) | 351 (63.5) | 63 (11.4) | 30 (5.4) |
| Health Professional (n=285) | 91 (31.9) | 174 (61.1) | 17 (6.0) | 3 (1.1) |
| Affected family member or friend (n=419) | 134 (32.0) | 227 (54.2) | 45 (10.7) | 13 (3.1) |

Table S1 b) Ratings for the statement “*The primary goal/purpose is clear*” broken down by end-user groups of Cracks in the Ice. A significant association between group and ratings on the 5-point Likert Scale were observed (*Χ*^2^_9_ = 43.79, *P* <0.01).

|  | **Strongly agree** | **Agree** | **Neither agree nor disagree** | **Disagree/Strongly Disagree** |
| --- | --- | --- | --- | --- |
| Community (n=809) | 393 (48.6) | 374 (46.2) | 26 (3.2) | 16 (2.0) |
| People who use ice (n=553) | 186 (33.6) | 307 (55.5) | 42 (7.6) | 18 (3.3) |
| Health Professional (n=285) | 117 (41.1) | 150 (52.6) | 16 (5.6) | 2 (0.7) |
| Affected family member or friend (n=419) | 176 (42.0) | 204 (48.7) | 30 (7.2) | 9 (2.1) |

Table S1c): Ratings for the statement “*The information and resources has been informed by evidence*” broken down by end-user groups of Cracks in the Ice. A significant association between group and ratings on the 5-point Likert Scale were observed (*Χ*^2^_9_ = 48.24, p <0.01).

|  | **Strongly agree** | **Agree** | **Neither agree nor disagree** | **Disagree/Strongly Disagree** |
| --- | --- | --- | --- | --- |
| Community (n=809) | 335 (41.4) | 403 (49.8) | 66 (8.2) | 5 (0.6) |
| People who use ice (n=553) | 158 (28.6) | 306 (55.3) | 73 (13.2) | 16 (2.9) |
| Health Professional (n=285) | 125 (43.9) | 141 (49.5) | 17 (6.0) | 2 (0.7) |
| Affected family member or friend (n=419) | 144 (34.4) | 227 (54.2) | 41 (9.8) | 7 (1.7) |

Table S1 d) Ratings for the statement “*The terminology is non-stigmatising*” broken down by end-user groups of Cracks in the Ice. A significant association between group and ratings on the 5-point Likert Scale were observed (*Χ*^2^_9_ = 52.72, p <0.01).

|  | **Strongly agree** | **Agree** | **Neither agree nor disagree** | **Disagree/Strongly Disagree** |
| --- | --- | --- | --- | --- |
| Community (n=809) | 311 (38.4) | 421 (52.0) | 69 (8.5) | 8 (1.0) |
| People who use ice (n=553) | 149 (26.9) | 305 (55.2) | 81 (14.6) | 18 (3.3) |
| Health Professional (n=285) | 111 (38.9) | 161 (56.5) | 12 (4.2) | 1 (0.4) |
| Affected family member or friend (n=419) | 141 (33.7) | 226 (53.9) | 45 (10.7) | 7 (1.7) |
