# Supplementary material for: Evaluation of a Digital Health Initiative in Illicit Substance Use: Cross-sectional Survey Study
Source: J Med Internet Res. 2021 Aug 10;23(8):e29026. doi: 10.2196/29026 (PMC8386404; doi:10.2196/29026)
Supplement: Multimedia Appendix 2 [file jmir_v23i8e29026_app2.docx]

### Multimedia Appendix 2

### Table S1: Examples of open-ended comments from study participants.

| **CITI visitors group** | **CITI naïve group** |
| --- | --- |
| Cracks in the Ice is a valuable resource that I personally use when working with families that are either affected by Ice or family members that are impacted by friends or family that use Ice. I find it to be user friendly for all ages and easy to navigate to find information required. Cracks in the Ice is the only comprehensive resource I have found to provide relevant informative which I also encourage work colleagues to use for their clients. | It's non-judgemental, and informative, not only for users, but for others around them who may be affected by their use. It is specific to this drug so there's no need to sift through other items to find what you are looking for. It's easy to navigate for the information you need |
| This resource is a clear, factual source of information. The public needs to hear the facts on what ICE actually is, and stop being so scared of it and people who use it. | I think it’s so important for people to have access to information no matter what it is Cracks on the ice seems to have really good intentions and genuinely want to help everyone no matter who they are Website was very easy to navigate and use, amazing work |
| Very useful, comprehensive website with unique breadth of information. Appreciate extensive information for different groups (friends, family, users, workers, schools) and that it is worded in non-judgmental and non-stigmatising ways | Cracks in the Ice is the best and most coherent source regarding ICE that I have seen and I would not hesitate in recommending it to a person in search of reliable and realistic information, with references clearly listed. |
| Resources are clear, free, and easily accessible with strong evidence-based background. | A good "one stop shop" |
| I found 'Cracks in the ice' very informative. A great tool for health professionals. | I love that there's also a Cracks in the Ice app available for free. This is good for both ice users and for their supporting families/friends. Plus I learned a lot on it myself! |
| helpful in my clinical work and useful to pass onto colleagues | Useful resource for users, family/friends of users, people at risk of becoming users |
| In my role as an AOD librarian I'm frequently asked for resources on methamphetamine for different population groups. I have consistently used and recommended Cracks in the Ice has a reliable evidence-based resource | If I knew someone using methamphetamines I would definitely recommend Cracks in the Ice. The website is clear, non-judgemental and informative |
| This resource provides a one-stop-shop for answering all the questions we get asked by persons who need help. We now have access tot a resource we can refer them to and importantly this includes a phone number with a real human on the other end who will take the call or at least return your call after leaving a message. The only extra info I would add is to make a section that builds on the fact that ice is just one drug, and it does carry the water for a number of other drugs that cause harms but don’t attract the same attention, such as synthetic cannabis and bath salts / flakka. Otherwise, keep up the good work and thank you! | This is the first website I have seen that has all this information in one spot. Unfortunately a lot of people I know who use ice already know most of the information they just think they don't have as bad a problem as they actually do. In saying that I would still recommend this website to everyone I know as it might help some of them open their eyes |
| When my friend was in a dark place it made me understand what he was going through and his road to recovery | There is lots of information, plenty of things I didn't know and useful contacts and resources. |
| I am unaware of any other website that would serve this purpose and support | Single source of info. Don’t have to dig. Acknowledges without judgement |
| I work in an Indigenous community some info is not useful or suitable | Could provide stories from people who have struggled with Ice, and interviews with people who have helped people struggling |
